# Bacterial–Fungal Interactions: Mutualism, Antagonism, and Competition

**DOI:** 10.3390/life15081242

**Published:** 2025-08-05

**Authors:** Manyu Zhang, Yuwei Zhang, Zhengge Zhao, Feilong Deng, Hui Jiang, Ce Liu, Ying Li, Jianmin Chai

**Affiliations:** 1 Guangdong Provincial Key Laboratory of Animal Molecular Design and Precise Breeding, School of Animal Science and Technology, Foshan University, Foshan 528000, China; manyuz9@163.com (M.Z.); 19503865010@163.com (Y.Z.); zhenggezhao@163.com (Z.Z.); fdeng@fosu.edu.cn (F.D.); jianghui1001@fosu.edu.cn (H.J.); 2 Institute of Animal Science and Veterinary Medicine, Shandong Academy of Agricultural Sciences, Jinan 250131, China; liuceshiyan@163.com

**Keywords:** interaction mechanism, microbiota, mutual benefit and symbiosis, antagonistic competition

## Abstract

The interaction between bacteria and fungi is one of the key interactions of microbial ecology, including mutualism, antagonism, and competition, which profoundly affects the balance and functions of animal microbial ecosystems. This article reviews the interactive dynamics of bacteria and fungi in more concerned microenvironments in animals, such as gut, rumen, and skin. Moreover, we summarize the molecular mechanisms and ecological functions of the interaction between bacteria and fungi. Three major bacterial–fungal interactions (mutualism, antagonism, and competition) are deeply discussed. Understanding of the interactions between bacteria and fungi allows us to understand, modulate, and maintain the community structure and functions. Furthermore, this summarization will provide a comprehensive perspective on animal production and veterinary medicine, as well as guide future research directions.

## 1. Introduction

Symbiosis, antagonism, and competition, the main interaction modes between bacteria and fungi, are widely presented in multiple ecological niches, such as the gut, rumen, skin, and other ecosystems. Mutualism is a win–win interactive model where bacteria and fungi achieve functional complementarity through resource sharing, which is crucial for maintaining ecosystem balance, promoting growth, and ensuring animal health. The probiotic approach, by supplementing beneficial microbial species into the host, can utilize this mutualistic relationship to regulate the balance of the microbiota and has been proven to be an effective alternative to antifungal agents [1]. However, bacteria and fungi do not always coexist in harmony. The phenomena of pathogen infection, antibiotic production, and biological control in antagonistic effects have important research and application value in the fields of agriculture and veterinary medicine. Meanwhile, the competition between the two in terms of resources and space maintains the stability of the microbial community through niche differentiation. An in-depth exploration of these interaction mechanisms between bacteria and fungi not only helps to reveal the ecological laws of microbial communities but also provides new ideas and strategies for solving practical problems, such as animal production, animal health maintenance, and disease treatment.

## 2. Mutualism

Mutualism is a win–win interaction mode where bacteria and fungi achieve functional complementarity through resource sharing, commonly observed in mycorrhizal symbiosis, lichen symbiosis, and animal skin microbiota [2]. These symbiotic relationships are critical for ecosystem stability, plant growth, and animal health.

It is well known that the gut microbiome plays a significant role in the health and disease of the host, and bacteria are the main microorganisms in the digestive system [3,4,5,6,7,8]. For instance, anaerobic gut fungi (e.g., Neocallimastigomycetes) are important members of the gut microbiome of herbivores [9]. In addition, anaerobic intestinal fungi, such as *Neocallimastix californiae* and *Anaeromyces robustus*, form mutualistic relationships with bacteria in the gut of mammals such as giant pandas, omnivorous carnivores, herbivores, and Yunnan golden monkeys [10]. These fungi are able to degrade cellulose in the rumen and produce secondary metabolites, such as antibiotics, that regulate the balance of the rumen microbial community [11,12].Intestinal fungi can also elicit deleterious effects on host health and have been associated with a number of disorders, including gastrointestinal diseases like inflammatory bowel disease (IBD), Crohn’s disease (CD), and colorectal cancer [13,14,15,16]. In CD, Candida tropicalis was overrepresented in patients and correlated positively with anti-*S. cerevisiae* antibodies [13,17]. In the Huntington’s disease (HD) intestinal fungi group, a variety of fungi such as *Glarea lozoyensis* and *M. restricta* were found to be strongly positively correlated with *Bacteroides* spp., *Prevotella scopos*, and other bacteria (*r* > 0.6) [17]. In the study of gut fungi and mice microbiome assembly, the presence of fungi affected the structure of bacterial communities, but the effect of bacteria on fungal communities was stronger and longer-lasting. Co-colonization of fungi and bacteria strongly altered the β diversity of fungal communities and significantly increased α diversity and richness. Moreover, the effects of fungal colonization on bacterial β diversity and richness were stronger at 4 weeks of age [18].

Bacterial–fungal symbiosis in animal skin microbiota (e.g., canine skin and ears) maintains barrier function and immune balance. Tang et al. analyzed 589 canine samples (257 ears and 332 skin) via next-generation sequencing (NGS), revealing diverse microbiota comprising Actinobacteria, Firmicutes, Proteobacteria, and fungi (e.g., Malassezia) in healthy dogs. Under healthy conditions, probiotics like *Lactobacillus* inhibit pathogens (e.g., *Staphylococcus pseudintermedius*) by secreting lactic acid and short-chain fatty acids, maintaining an acidic pH (4.5–5.5). A study showed *Lactobacillus* abundance in healthy canine skin reduces *Malassezia* overgrowth risk [19]. In diseased states (e.g., atopic dermatitis, otitis externa), microbial diversity declines, with *S. pseudintermedius* and *Malassezia* dominating and disrupting symbiosis. However, certain bacteria (e.g., *Bacillus*) restore balance by secreting antifungal compounds (e.g., bacilysin) [19]. *Phomopsis liquidambari* is an endophytic fungus with a wide host range, which was initially isolated from the inner bark of *Bischofia polycarpa*. The colonization of *P. liquidambari* in rice plants can shape the bacterial community structure by altering the rhizosphere microenvironment (the interface for nutrient exchange between plant roots and soil, such as the secretions of symbiotic plant roots and oxygen), or through autocrine substances, promoting and inhibiting various local soil microorganisms to achieve better nitrogen and phosphorus functional levels, increasing the structural and functional diversity of microorganisms in the same paddy soil, and providing a better survival environment for most local soil microorganisms [20].

## 3. Antagonism

Antagonism involves reciprocal inhibition between bacteria and fungi, including pathogen infection, antibiotic production, and biological control, with wide applications in agriculture and veterinary medicine. There is substantial evidence that *Candida albicans* in the gastrointestinal tract is a major source of systemic candidiasis [21,22,23]. *Candida albicans* is pathogenic because it changes its morphology from yeast to hyphae after adhering to host cells, enabling the fungus to invade host cells, cause damage, and cross the barrier for translocation [24]. Studies have shown bacterial metabolites, such as fatty acids, can inhibit *C. albicans* hyphal formation [25,26], and *Lactobacillus rhamnosus* significantly shortened the length of *Candida albicans* hyphae (by 42%) and inhibited *Candida albicans* by reducing its filamentation and translocation [24]. *C. albicans* can support the survival of *Clostridioides difficile* under aerobic conditions, while *C. difficile* inhibits *C. albicans* filamentous growth [27]. *Microsporum canis,* a major dermatophyte in dogs and cats, was analyzed by Wang et al. via genome and transcriptome comparisons between invasive dermatophytosis (ID) and tinea capitis (TC) strains [28]. ID strains upregulated protease (e.g., subtilisin), lipase, and heat shock protein (HSP70, HSP90) genes at 37 °C, enhancing tissue invasion and heat tolerance. TC strains overexpressed adhesion proteins and urease at 28 °C, promoting keratin degradation and hair shaft attachment [28]. Aneke et al. further found *M. canis* strains from lesioned hosts exhibited higher lipase and catalase activity, aligning with ID virulence traits [29]. These mechanisms indicate bacteria and fungi exacerbate host damage synergistically or independently. Antibiotic production is a core bacterial antifungal mechanism. *Bacillus velezensis*, for example, secretes bacilysin, a broad-spectrum antifungal peptide that inhibits chitin synthase, disrupting cell wall synthesis and increasing permeability. In Botrytis cinerea, bacilysin reduces chitin content, causing hyphal swelling and growth arrest. It also chelates zinc ions, inhibiting key enzymes like pyruvate dehydrogenase, reducing ATP production and hyphal elongation. In line with this, several studies have shown the opposing relationship between *Lactobacillus reuteri* and fungal colonization [18,30,31]. Reuterin, a main metabolite secreted by *L. reuteri*, is a well-known antimicrobial substance that can also suppress fungal growth. Additionally, lactic acid and organic acids are also products of *L. reuteri* that could decrease gut pH and impact fungal growth. Apart from that, *L. reuteri* has neuromodulatory effects in the enteric nervous system since it is capable of producing the neurotransmitter gamma-aminobutyric acid (GABA), which could also directly impact the growth of other residents in the gut [31,32,33]. Given that a delicate bacteriome–mycobiome balance is required to maintain host health and homeostasis, the altered interkingdom equilibrium in HD could contribute to or modulate gastrointestinal inflammation or the microbiome–gut–brain axis in HD.

In addition, *Saccharomyces boulardii* is widely used as probiotics because they secrete enzymes that inactivate toxins produced by residents of the inflamed gut and inhibit the growth of other potential pathogens [34,35,36]. For example, *Saccharomyces boulardii* is a non-pathogenic yeast that is commonly used for the prevention and treatment of *Clostridium difficile*-associated diarrhea and colitis [37]. Clostridium difficile toxin is a key factor leading to intestinal lesions. It can trigger a series of pathological responses after binding to brush border receptors. Saccharomyces boulardii reduces part of the enterotoxic effects of the toxin by inhibiting the binding of the toxin to its receptor, which is mediated by the secretion of proteases by yeast [38]. *Bacillus velezensis JW* demonstrated significant probiotic potential through genomic analysis. Its genome contains four fungicin gene clusters, three polyketo synthase (PKS) gene clusters, and five non-ribosomal peptide synthase (NRPS) gene clusters. The antibacterial metabolites encoded by these genes (such as lipopeptides and polyketone compounds) may have inhibitory effects on fungal pathogens [39]. Although this study mainly verified the broad-spectrum antibacterial activity of JW against pathogenic bacteria in fish, such as *Aeromonas hydrophila* and *Vibrio parahemolyticus*. However, *B. velezensis* strain is known to be able to destroy the fungal cell membrane through liposeptides (such as surfactin, iturin, and fengycin) and inhibit plant and animal fungal pathogens such as *Fusarium* and *Alternaria* [40]. For example, iturin and fengycin can interfere with the synthesis of β-1, 3-glucan in the fungal cell wall, leading to cell rupture. The presence of NRPS and PKS genes in the JW genome suggests that it may have similar antifungal capabilities, although further experimental verification is needed. Combined with the characteristics of JW in enhancing immunity and disease resistance in crucian carp (Carassius auratus), its antifungal metabolites are expected to provide a biological control strategy alternative to antibiotics for aquaculture.

Moreover, there is another good example of probiotics of fungus. The application of *Bacillus velezensis* Y01, as a probiotic in the feed of Langya chickens, shows significant growth-promoting and immune-enhancing effects [41]. During the 42-day experiment, the addition of feed with 2.0 × 10^9^ CFU/kg Y01 increased the body weight of Langya chickens by 7.01% and the daily weight gain by 7.24% (*p* < 0.05), and by increasing the abundance of beneficial bacteria (such as Firmicutes and Bacteroidetes) and reducing harmful bacteria, the structure of the cecal microbiota improves and the colonization of pathogenic bacteria is indirectly reduced. The *B*. *velezensis* strain is renowned for its encoded antibacterial metabolites, such as lipopeptides surfactin, iturin, and polyketo compounds, which can disrupt fungal cell membranes and inhibit plant and animal fungal pathogens like Fusarium and *Alternaria* [40]. The genome of Y01 may contain similar non-ribosomal peptide synthase (NRPS) and polyketo synthase (PKS) gene clusters, suggesting its antifungal potential, especially in reducing intestinal fungal pathogens in poultry such as *Aspergillus*. Y01 enhances immune function by increasing the levels of serum IgG (36.09%), IgM (56.08%), and IL-2 (32.83%) (*p* < 0.05), and may further inhibit fungal infections [41]. These characteristics indicate that Y01, as a probiotic, not only improves the growth and intestinal health of chickens but also may provide a biological control strategy alternative to antibiotics for poultry farming through antifungal metabolites.

**Figure 1:**
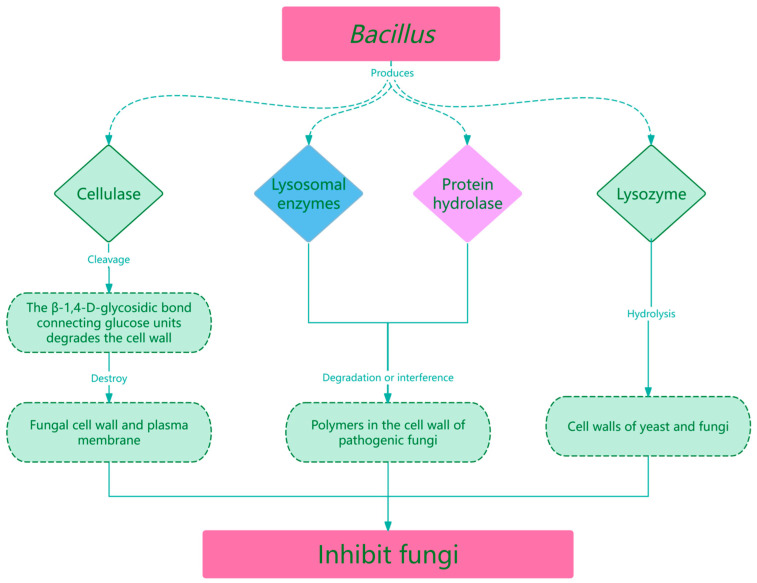
Mechanisms by which Bacillus inhibits fungi.

*Aspergillus* and *Trichophyton mentagrophytes* are among the most important fungal pathogens [42]. *Aspergillus fumigatus* can thrive in almost any organic matter and exists in various habitats, including soil, air, and water [43]. *Aspergillus fumigatus* and *Aspergillus niger* are the etiologies of infections such as aspergilloma, allergic bronchopulmonary aspergillosis, chronic pulmonary aspergillosis, and invasive aspergillosis. *Trichophyton mentagrophytes* is an important agent of zoonotic fungal infections, which can be transmitted from asymptomatic pets to humans [44]. Studies have shown that *Bacillus subtilis* inhibits *Aspergillus niger* more significantly than *Aspergillus fumigatus*, while *Bacillus axarqueinsis* is more effective against *Aspergillus fumigatus* than *Aspergillus niger*. *Bacillus licheniformis* and *Bacillus subtilis* show approximately threefold-higher inhibition percentages against two *Trichophyton* species than against *Aspergillus fumigatus* and *Aspergillus niger* on plates. However, in liquid cultures, *Bacillus subtilis* exhibits higher fungal inhibition percentages against *Aspergillus fumigatus* than against *Trichophyton* [45]. The genus *Bacillus* antagonizes fungi by affecting enzyme activity. Cellulases degrade the cell wall by cleaving the ß-1, 4-D-glycosidic bond connecting the glucose unit containing cellulose and exert antifungal effects by destroying the fungal cell wall and cytoplasmic membrane. Lysosomes and proteolytic enzymes inhibit fungal growth and differentiation by solubilizing or interfering with polymers in the cell wall of pathogenic fungi. Lysozyme inhibits fungi by hydrolyzing their cell wall [46]. The specific mechanism is shown in Figure 1.

## 4. Competition

Competitive interactions involve resource or spatial rivalry between bacteria and fungi, maintaining community stability via niche differentiation. Molecular mechanisms by which fungi regulate bacterial fitness through resource competition and chemical antagonism, such as antibiotics. Through genomic-level analysis of 16 bacteria–fungal pairings (involving 8 fungi and 2 bacteria isolated from the microbiota of cheese skins), researchers found the complex competitive interactions between fungi and bacteria in the microbiota of cheese skins [47]. Iron is a key driver of competition between fungi and bacteria [48]. Using random barcode transposon site sequencing (RB-TnSeq), the study found that fungi significantly altered the adaptability of bacterial mutants, especially in terms of the acquisition of iron and biotin. All fungi compete for the limited iron resources in the environment by generating iron chelators (such as siderophores) [47], leading to a decline in the adaptability of mutants of bacterial iron metabolics-related genes (such as tonB, entF). Furthermore, some fungi inhibit bacterial growth through secondary metabolites (e.g., antibiotics). For instance, penicillin compounds produced by *Penicillium* significantly reduce the survival rate of *Escherichia coli* mutants (*p* < 0.05). The research also found that fungus-induced biotin deficiency further intensified competition and affected the adaptability of mutants in bacterial biotin synthesis genes (such as bioB). These competitive interactions not only shape the structure of the cheese skin microbiota but also may indirectly promote the ecological advantages of fungi by restricting bacterial growth. Experiments on knocking out regulatory genes of fungal secondary metabolites (such as laeA) have shown that secondary metabolites are the core drivers of competitive interactions [47].

In a healthy gut, diverse bacterial communities suppress *Candida albicans* overgrowth through competitive exclusion and production of antibacterial substances like LL-37, as evidenced by mouse model studies showing resistance to fungal colonization. Antibiotics disrupt this balance, reducing anaerobic bacteria and enabling *C. albicans* to proliferate, particularly in immunocompromised individuals. Probiotics, such as *Lactobacillus rhamnosus*, counteract this by inhibiting fungal adhesion and hyphal formation via metabolic byproducts like fatty acids. Additionally, *C. albicans* engages in complex interspecies competition, supporting *Clostridioides difficile* survival under aerobic conditions while *C. difficile* inhibits its hyphal growth, as well as reducing *Pseudomonas aeruginosa* virulence by suppressing toxin production [36]. These competitive dynamics highlight the critical role of a balanced microbiome in controlling *C. albicans* and preventing its shift to an opportunistic pathogen. In alcoholic fermentation, yeast (such as *Saccharomyces cerevisiae*) competes with lactic acid bacteria for nutrients, affecting the growth of lactic acid bacteria and the fermentation process. The competition influences the quality and flavor of the fermented products [49]. In the rumen system of ruminants, the feed additive *Saccharomyces cerevisiae* (*S. cerevisiae CNCM I-1077*) competes with rumen bacteria for nutrients, affecting the dynamics of the microbiota and the digestive efficiency of ruminants [49]. The rumen is the most important digestive organ in ruminants [50] and in the human microbiota. It contains a large number of eukaryotic microbes and bacteria [8,51,52]. *Pseudomonas aeruginosa* in the intestine kills or inhibits the mycelium formation of *Candida albicans* by secreting quorum-sensing molecules, such as phospholipase C and phenazine [53]. Lactic acid bacteria (such as *Lactobacillus acidophilus*) inhibit the growth of *Candida albicans* through the peroxide system. Oral *Streptococcus* and *Candida albicans* compete for adhesion sites through polysaccharide receptor-mediated adhesion in the oral cavity [53]. Bacteria and fungi in biofilms compete for space and resources within the biofilms, influencing the development of infectious diseases. These competitive interactions maintain the balance of the microbiota in a healthy state, but an imbalance (such as after the use of antibiotics) may lead to infections [54].

## 5. Conclusions

There are three kinds of interactions between bacteria and fungi, including symbiosis, antagonism, and competition. Mutualism, which is widely found in mycorrhizae, lichens, and animal microbiota, promotes the growth of plants and animals, while excessive proliferation of some fungi can also threaten the health of the host. For example, bacterial metabolites in the gut can inhibit the mycelium formation of *Candida albicans*, and bacilysin secreted by *Bacillus velenzensis* can be used in agricultural biological control, aquaculture, and poultry breeding. The competition focuses on resources and space, such as iron and biotin, which affects the structure of microbial communities and maintains the balance of microbial communities in the gut, fermentation system, and other environments. These interactions are of great significance to ecosystem stability, host health, and agricultural production, which provide an important theoretical basis for multi-field research and application.

With advancing technology, breakthroughs in bacterial–fungal interactions are anticipated, but not without technical and conceptual hurdles. Single-cell sequencing and spatial transcriptomics, hailed for high-resolution analysis, face practical constraints: single-cell isolation from complex matrices (e.g., soil, gut mucosa) remains inefficient, and spatial transcriptomics struggle to capture transient interactions (e.g., short-lived metabolite exchanges), risking oversimplification of dynamic networks.

Interdisciplinary cooperation will become an important trend in research. By integrating synthetic biology techniques, gene editing of bacteria and fungi can be carried out, and artificial synthetic microbiota can be designed to achieve precise regulation of specific functions, such as customized biological control agents or environmental remediation microbiota. From the perspective of ecological economics, the value of microbial interactions in ecosystem services can be evaluated to provide more scientific decision-making basis for industries such as agriculture and environmental protection. Future research directions include deeply dissecting the molecular mechanisms of interactions between *Microsporum canis* and *Bacillus velenzensis* through integrated multi-omics technologies (e.g., genomics, transcriptomics, metabolomics) [55], developing and optimizing Bacillus-based biocontrol strategies for expanding their applications in veterinary clinics against *M. canis* infections and agricultural disease management [29]. Captive giant pandas are prone to gastrointestinal diseases. Frequent use of antibiotics will lead to increased antibiotic resistance and the emergence of antibiotic resistance genes (ARGs), which will aggravate the health risks of this endangered population. Based on the mechanism of interaction between fungi and bacteria, corresponding probiotics are developed to replace antibiotics and protect the health of panda population [56,57]. Probiotics developed to replace antibiotics in giant pandas may face host specificity issues: gut microbiomes of captive pandas differ drastically from wild populations, and lab-tested probiotics often lose efficacy in semi-natural enclosures [56]. Harnessing the gut–skin axis to develop novel therapies for fungal-caused skin diseases [58].

In terms of practical application, in response to the current drug resistance crisis caused by the abuse of antibiotics, the development of new biological agents based on the antagonistic mechanism between bacteria and fungi, such as targeted antimicrobial peptides and prebiotic combinations, is expected to become a green solution to replace traditional antibiotics. Exploring the utilization of microbial interactions to regulate human microecology and developing personalized microecological regulators will bring brand-new strategies for disease prevention and treatment.

## Data Availability

No new data were created or analyzed in this study. Data sharing is not applicable to this article.

## Abbreviations

The following abbreviations are used in this manuscript:

IBD	Inflammatory bowel disease
CD	Crohn’s disease
*S. pseudintermedius*	*Staphylococcus pseudintermedius*
*C. albicans*	*Candida albicans*
*C. difficile*	*Clostridioides difficile*
*M. canis*	*Microsporum canis*
TC	Tinea capitis
ID	Invasive dermatophytosis
HD	Huntington’s disease
*B. velezensis*	*Bacillus velezensis*
*L. reuteri*	*Lactobacillus reuteri*
GABA	Gamma-aminobutyric acid
NRPS	Non-ribosomal peptide synthase
PKS	polyketo synthase
RB-TnSeq	Random barcode transposon site sequencing
*P. liquidambari*	*Phomopsis liquidambari*
ARGs	Antibiotic resistance genes
